# Supplementary material for: Content-rich biological network constructed by mining PubMed abstracts
Source: BMC Bioinformatics. 2004 Oct 8;5:147. doi: 10.1186/1471-2105-5-147 (PMC528731; doi:10.1186/1471-2105-5-147)
Supplement: Additional File 5 — The original Chilibot query results of the term "long-term potentiation (LTP)" and 22 other terms, limiting the latest references analyzed to the years 1990, 1995, 2000, and 2004. [file 1471-2105-5-147-S5.bz2 › chilibotAdditionalFile5/ltp2000/html/TRKB_PKC.html]

 


 **TRKB** and **PKC** 
  
Found 2 abstracts in PubMed,  **2 abstracts were retrieved and analyzed**.  


---

 Search Google  |
 PDF files only 
|  EDU domain only 

---

- J Neurochem, 1995   **Characterization of **TrkB** receptor mediated signaling pathways in rat cerebellar granule neurons involvement of protein kinase C in neuronal survival.** .
  **TrkB** belongs to the Trk family of tyrosine kinase receptors and mediates the response to brain derived neurotrophic factor BDNF and neurotrophin 4 5 NT 4 5 .
  Here, we report that both truncated and full length forms of **TrkB** receptors are expressed in developing cerebellar granule neurons.
  BDNF and NT 4 5 increased the survival of cultured cerebellar granule neurons.
  BDNF and NT 4 5 also induced an autophosphorylation of **TrkB** receptors and subsequently resulted in a phosphorylation and binding of phospholipase C gamma PLC gamma and SH2 containing sequence to the autophosphorylated **TrkB** receptors.
  Both contain src homology 2 SH2 regions.
  In keeping with a signaling function of PLC gamma, BDNF increased the phosphatidylinositol PI turnover and elevated intracellular calcium levels.
  To investigate the involvement of protein kinase C **PKC** in the survival of granular neurons, we show here activation of **PKC** after BDNF or TPA treatment and blocking of the observed survival promoting effects of BDNF and TPA with calphostin C, a specific **PKC** inhibitor.
  In addition, BDNF activated c ras in a concentration dependent manner.
  These results suggest that two different pathways, the c ras and the PLC gamma pathway, are activated by **TrkB** receptors in primary neurons.
  **PKC** activation is involved in the survival promoting effect of BDNF.
